# Supplementary material for: Ticks Infesting Dogs in Khyber Pakhtunkhwa, Pakistan: Detailed Epidemiological and Molecular Report
Source: Pathogens. 2023 Jan 6;12(1):98. doi: 10.3390/pathogens12010098 (PMC9862609; doi:10.3390/pathogens12010098)
Supplement: Supplementary file 1 [file pathogens-12-00098-s001.zip › pathogens-2016204-supplementary.pdf]

**Table S1. Ixodid ticks' *cox1* partial nucleotide sequences (query) NCBI GenBank BLAST summary**

| Khyber Pakhtunkhwa Pakistan (Present study) | | NCBI GenBank Published Report (Global Record) | | |
|---|---|---|---|---|
| **Tick species** | **Query Sequences (Acession No.)** | **Subject Sequences (Acession No.)** | **BLAST similarity** | **Country** |
| *Rh. sanguineus s.l.* | ON911972 | MG969507 | 100% | China |
| *Rh. sanguineus s.l.* | ON911973 | | | |
| *Rh. sanguineus s.l.* | ON911974 | | | |
| *Rh. sanguineus s.l.* | ON911975 | | | |
| *Rh. turanicus s.s.* | ON911976 | MT800313 | 100% | Pakistan |
| *Rh. turanicus s.s.* | ON911977 | MW642242 | 99.79% | |
| *Hy. dromedarii* | ON911978 | KT920181 | 100% | Iran |
| *Hy. excavatum* | ON911979 | MW546284 | 100% | Turkey |
| *Rh. microplus* | ON911980 | MK462194 | 100% | Pakistan |
| *Rh. haemaphysaloides* | ON911981 | MT800317 | 100% | Pakistan |
| *Rh. haemaphysaloides* | ON911982 | | | |

**Table S2. Ixodid ticks, *16S rRNA* partial nucleotide sequences (query) NCBI GenBank BLAST summary**

| Khyber Pakhtunkhwa Pakistan (Present study) | | NCBI GenBank Published Report (Global Record) | | |
|---|---|---|---|---|
| **Tick species** | **Query Sequences (Acession No.)** | **Subject Sequences (Acession No.)** | **BLAST similarity** | **Country** |
| *Rh. sanguineus s.l.* | ON921112 | MG651947 | 100% | China |
| *Rh. sanguineus s.l.* | ON921113 | | | |
| *Rh. sanguineus s.l.* | ON921114 | | | |
| *Rh. sanguineus s.l.* | ON921115 | | | |
| *Rh. sanguineus s.l.* | ON921116 | | | |
| *Hy. excavatum* | ON921119 | MT229183 | 100% | Turkey |
| *H.y dromedarii* | ON921120 | KU130425 | 100% | Senegal |
| *Rh. haemaphysaloides* | ON921121 | MZ436881 | 100% | Pakistan |
| *Rh. haemaphysaloides* | ON921122 | | | |
| *Rh. microplus* | ON921123 | MK495912 | 100% | Pakistan |
| *Rh. turanicus s.s.* | ON921124 | MT799955 | 100% | Pakistan |
| *Rh. turanicus s.s.* | ON921125 | MT799954 | | |
